# Development of motivational self-regulation in childhood—An integrative review

**DOI:** 10.3389/fpsyg.2025.1533625

**Published:** 2025-06-13

**Authors:** Maike Trautner, Martin Pinquart

**Affiliations:** Developmental Psychology, Department of Psychology, Philipps University of Marburg, Marburg, Germany

**Keywords:** motivational self-regulation, self-regulated learning, development of self-regulation, motivational development, integrative review

## Abstract

Motivational self-regulation is an important skill supporting task engagement, achievement, and wellbeing during learning and other potentially strenuous tasks. It refers to active thoughts and behaviors used to initiate or maintain one's task engagement by manipulating underlying motivational processes (Wolters, 2003). This involves knowledge about one's own motivation and task characteristics, motivational control strategies, as well as monitoring of motivational processes. Most research on motivation regulation has focused on adolescents and adults; but little is known about motivation regulation in pre- and primary school children and its development. The present integrative review therefore analyzes theoretical accounts of motivation regulation to identify components of the process which may develop during childhood. To draw tentative hypotheses on how the complex process develops during childhood, it reviews research on the development in related areas of self-regulation, for example, self-regulated learning and emotion regulation. Drawing on this, it poses questions for future research on assessment methods, the development of metamotivational knowledge, monitoring, and control, as well as factors influencing their development.

## 1 Introduction

Motivational self-regulation, defined as the active use of thoughts and behaviors by which individuals intentionally increase their willingness to begin or complete a task/goal-achievement process, plays an important role in the achievement of personal goals, performance, and wellbeing (e.g., Fong et al., 2024; Miele and Scholer, 2018; Schwinger and Stiensmeier-Pelster, 2012; Wolters, 2003). Most studies on motivation regulation have been conducted in an academic context with school and college samples in cross-sectional designs (Trautner et al., 2025). Thus, little is known about how people acquire motivation regulation in childhood, how it develops from childhood to adulthood, and how early motivation regulation affects academic and wellbeing related outcomes later in life—despite calls for this (Miele et al., 2024; Miele and Scholer, 2018; Wolters, 2003). Existing research and theoretical frameworks regarding the development of motivation regulation in childhood are scarce and scattered across several related domains, such as volitional control, emotion regulation, metacognition and self-regulated learning. The present paper seeks to review and integrate theoretical approaches describing motivational self-regulation and the development of self-regulation in related domains to build a heuristic theoretical framework as a basis for future investigations on the development of motivational self-regulation during childhood. A better understanding of the development and conditions under which children acquire motivation regulation skills can help to develop appropriate assessment methods for different age groups, to spark future research and to implement effective support measures at an early stage in order to counteract persistent motivational (regulation) problems. To this end, we first derive components of the motivation regulation process from existing theories on motivation regulation which may be subject to development, as well as the developmental preconditions they imply. Second, we discuss the development of motivation regulation components and factors influencing their development, including mechanisms by which children acquire motivation regulation skills. Here, we draw on theories and findings from related domains of self-regulation with conceptual overlap to motivation regulation, such as emotion regulation, as they imply similar developmental processes. Finally, we pose predictions and questions for future research on the development of motivation regulation. Table 1 provides an initial summary of propositions from several theoretical strands with respect to core aspects of the motivation regulation process, while Table 2 focuses on specific motivation regulation strategies as one aspect of the process derived from these theoretical perspectives, including their developmental prerequisites.

**Table 1:** Comparison of theoretical foundations of motivation regulation.

		**A) Process model of motivation regulation (Schwinger and Stiensmeier-Pelster, 2012)**	**B) Metamotivational model of motivation regulation (Miele and Scholer, 2018; Miele et al., 2024)**	**C) Motivational theory of lifespan development (Heckhausen and Schulz, 1995; Heckhausen et al., 2010)**
1)	Theories, frameworks, and ideas the models/theories build on	•Models of self-regulated learning (Pintrich, 2004) •Definition of motivation regulation (Wolters, 2003) •Cybernetic models of self-regulation (Carver and Scheier, 1990) •Interest regulation (Sansone and Thoman, 2005)	•Existing models on motivation regulation (Sansone and Thoman, 2005; Schwinger and Stiensmeier-Pelster, 2012) •Definition by Wolters (2003) •Metacognition frameworks (e.g., Nelson and Narens, 1990) •Theories of self-regulated learning, volitional control, and metacognition (Boekaerts, 1995; Corno, 1993; Kuhl, 2000; Pintrich, 2004)	•Life span theories of development (Baltes, 1987) •Control-related theories and theories of coping, and assimilation and accommodation processes (e.g., Brandtstädter and Renner, 1990; Folkman et al., 1986; Rothbaum et al., 1982) •Action phase model of developmental regulation (Wrosch and Heckhausen, 1999)
2)	Occurrence of motivational problems	•When a motivational level too low to continue a task is perceived (see monitoring) •Motivational problems are not uniform, but may differ in their quality (tasks being difficult or boring) and quantity	•Result from a lack of motivation (quantitatively) or experiences of the wrong type of motivation (qualitative), or a discrepancy between current task goal motivation and a superordinate goal are perceived •Regarding quality, different motivational components are identified which relate to specific sets of experiences	•When environmental, societal, or personal circumstances do not match personal needs and goals
3)	Motivational monitoring	•By observing a need for higher motivation (e.g., if the task is done due to reasons beyond intrinsic joy in doing it, such as rewards and punishments, personal value attached to the 4 (task/goal, should or ought principles, goals) •Monitoring the magnitude of discrepancy between desired and current motivation •Attributing reasons to motivational problems	•Monitoring occurs with respect to the quantity and quality of motivation in relation to a task at hand •Different motivational components co-occur with different metamotivational feelings signaling specific qualities of motivational problems (facilitating attribution of the type of problem) •Can be monitored by signals from the object level of monitoring (bottom-up-monitoring) or monitored intentionally via executive processes checking on current motivational experiences in relation to demands (top-down monitoring) •Development of superordinate goals important to experience discrepancies between current and desired motivation	•No explicit mentioning of situational monitoring processes •Striving for action-outcome contingencies and expectations about such action-outcome-contingencies is central for goal engagement and disengagement and can be considered as trans-situational monitoring or metamotivational knowledge about oneself, the task, and regulation strategies
4)	Motivation to apply motivational control	•No explicit mentioning of motivation to apply control beyond the perceived need for higher motivation •Suggests that a “threshold” may be necessary to apply motivation regulation strategies	•Discusses whether motivation regulation may fail in spite of sufficient knowledge and ability for motivational control, but a lack of desire to regulate •Agency-related beliefs to regulate motivation (self-efficacy for motivation regulation, malleability theories about motivation) support strategy application	•Fundamental assumptions: humans seek primary control (creating event-behavior-contingencies and avoiding losses of these) •Suggests “functional primacy” of primary over secondary control, and compensatory effects of secondary control if primary control is not possible
5)	Application of motivation regulation strategies	•Suggests application of motivation regulation strategies to change and maintain motivation •Tentative mentioning of strategies being more effective when applied in correspondence to matching motivational problems and magnitude of the motivational problem •Quality of motivation regulation strategy application is important for their effectivity	•Suggests application of motivation regulation strategies to change and maintain motivation •Strategies are selected in response to and matching the type of motivational problem encountered	•Differentiate primary control (individuals modify the environment to meet their needs) and secondary control (strategies aimed at changing the self, one's own actions, thoughts, and preferences, to match environmental demands) •Strategy selection is a function of environmental constraints and opportunities, as well as goals •Shifts between primary and secondary control are possible
6)	Role of individual factors	•Moderator variables affecting the frequency and effectivity of motivation regulation strategy use •Cognitive abilities, prior knowledge, motivational dispositions, personality characteristics	•Metamotivational knowledge impacts of effectively motivational monitoring and control processes are executed, including task knowledge, strategy knowledge, and self-knowledge •Individual factors potentially influencing acquisition: interoception, emotion differentiation, trust in feelings as information	•Individual capacities for primary and secondary control exertion, e.g., individual agency
7)	Role of contextual factors	•Moderator variables affecting the frequency and effectivity of motivation regulation strategy use •Examples: task and subject characteristics, setting (homework, schoolwork)	•Motivational task demands and characteristics afford specific types of motivation and thus, which strategies are most effective	•Environmental conditions are relevant via constrains and opportunities they provide for primary or secondary control striving (e.g., direct physical environment, biological conditions; societal norms, …)
8)	Additional claims with respect to development	•No explicit developmental claims are made	•Suggests that older students may have more accurate motivational monitoring abilities than younger children •Development of motivation regulation may depend on executive function, metacognition, and theory of mind •Both beliefs about motivation and motivation regulation are acquired, e.g., via learning from role models, logical reasoning, trial-and-error learning	•Capacities for primary and secondary control change over the life course •Primary control capacity increases over the first years well into midlife •Secondary control striving (and capacity) develop slightly delayed to compensate limitations to primary control possibilities
9)	Adaptive motivation regulation and age appropriateness	•Strategies are not universally adaptive, but need to match the underlying motivational problem	•Strategies are not universally effective and adaptive, but need to match the underlying motivational problem	•Primary control enables a maximum of personal development and thus has functional primacy over secondary control, especially at younger ages •Secondary control is adaptive when primary control is not feasible •Primary control can be maladaptive if contingencies between action and outcome are illusory •Some secondary control strategies are labeled as dysfunctional (e.g., self-handicapping) •Functionality of strategies cannot be derived from the strategy itself, but depends on whether it matches the situation and long-term consequences

**Table 2:** Motivation regulation strategies and developmental preconditions.

**Motivation regulation strategy**	**Sample references for each strategy**	**Sample thought or behavior**	**Psychological mechanism by which the strategy motivates; primary vs. secondary control**	**Reaction to which motivational problem?**	**Developmental preconditions and empirical evidence**
Environmental control	Miele and Scholer (2018); Schwinger et al. (2007); Wolters (1998, 1999)	I try to eliminate all possible distractions before I start studying.	Behavioral strategy, influencing or selecting a learning environment to make learning more appealing or less strenuous Primary control	Low task value, low success expectancies, reducing costs and enhancing task focus due to distractions from the environment	•Experiencing distractions and the associated burden as unpleasant (costs) •Understanding that environmental distractions can impede goal progress •Physical ability to remove distractions (motor ability and environmental degrees of freedom) • Cooper and Corpus (2009): 6-year-olds mention environmental control as an adequate strategy to enhance motivation and give adequate reasons for doing so → 5 years?
Regulation of relatedness	Engelschalk et al. (2015); Norouzi et al. (2021); Schwinger et al. (2007)	I learn together with others to motivate myself.	Behavioral strategy, enhancing psychological need of relatedness Primary control	Need satisfaction, enhancing social relatedness	•Understanding of feeling of relatedness as a facilitator to intrinsic motivation/need frustration regarding relatedness as an impediment to motivation → 6–10 years?
Self-consequating	Miele and Scholer (2018); Schwinger et al. (2007); Wolters (1998, 1999)	I think of a reward that I give myself when I've finished learning.	Behavioral and mental strategy, self-inducing extrinsic incentives for completing an action Secondary control	Low task value, enhancing extrinsic/external motivation	•Monitoring of feelings and appraisals associated with low task value/low intrinsic motivation, e.g., boredom, discontentment •Understanding that rewards make task completion more appealing • Gurland and Glowacky (2011): rewards are seen as a helpful strategy to increase task interest in 8-year-olds • Cooper and Corpus (2009): 6-year-olds mention self-consequating as a helpful strategy to increase motivation, but do not yet give adequate psychological explanations why, this is the case for 10-year-olds → around 6 years?
Enhancement of introjected motivation	Miele and Scholer (2018); Schwinger et al. (2007)	I tell myself that I will feel guilty if I don't study now.	Mental strategy of visualizing the consequences of (non-) action, making clear the incentive of the consequences, what happens if an action result is not achieved Secondary control	regulation of introjected motivation, enhancing utility value and incentive for consequences of actions	•internalized standards for behavior or achievement of others •need and willingness to comply with others' standards •strongly dependent on the respective behavior and normative standard for it → 6–8 years?
Efficacy self-talk	Engelschalk et al. (2015); Miele and Scholer (2018)	If I don't think I can do the task, I tell myself, “You can do it!”.	Mental strategy, self-instruction to increase the expectation of self-efficacy by remembering previous successes and skills (Bandura, 2006: mastery experiences), increasing the fulfillment of basic psychological needs—experience of competence Secondary control	Low success expectancies, enhancing self-efficacy and perceived competence	•Concept of success expectancy, task-related abilities, fear of failure •Self-talk abilities •Memory of prior successes and failures •More or less realistic estimations of own future success → 6–8 years?
Enhancement of situational interest	Miele and Scholer (2018); Schwinger et al. (2007); Wolters (1998, 1999)	I turn studying into a game.	Mental and behavioral strategy, altering the task or task appraisals to include more intrinsically appealing and interesting elements Secondary control	Low task value, enhancing intrinsic value/interest	•Feelings of boredom, understimulation and discontentment, separation of goals and means of goal achievement, negative feelings attributed to means, not to goal achievement • Cooper and Corpus (2009): 6- and 8-year-olds hardly mention this strategy, about 50% of 10 year-olds do, but do not yet give adequate explanations why the strategy is useful. → 10–12 years?
Proximal goal setting	Miele and Scholer (2018); Schwinger et al. (2007); Wolters (1998, 1999)	I break down my learning into small pieces so that I feel I can manage it more easily.	Mental strategy, setting smaller, more reachable (and thus potentially more attractive) goals, lowering the level of construal Secondary control	Low task value and low success expectancies, enhancing personal significance/utility value, enhancing success expectancy	•Experiences of frustration; fear of failure; feeling overwhelmed as an experience of low success expectancy as a motivational problem •Ability to metacognitively analyze a task and plan ahead smaller steps to reach this •To some extent visible as metacognitive strategy use in 5-to-7-year-olds (Bryce and Whitebread, 2012) → 8–10 years?
Mastery self-talk	Schwinger et al. (2007); Wolters (1998, 1999)	I make myself realize that my goal is to learn as many new things as possible.	Mental strategy, activating the personal higher order goal of gaining competence, activating criterial and individual reference norms to set a goal standard Secondary control	Low task value (intrinsic value and attainment value)	•Experiencing joy and curiosity while exploring new things and acquiring skills •Using temporal, intra-individual reference norms to evaluate one's own progress •Personal higher order goal representation, mastery goal for the respective task or content, mastery goal orientation • Cooper and Corpus (2009): 8 year-olds mention this strategy frequently to the same amount as older learners, including adults, but neither age group gave adequate reasons why this strategy is useful. → 8–10 years?
Performance-approach self-talk	Miele and Scholer (2018); Schwinger et al. (2007); Wolters (1999)	I remind myself that I want to do better than the others on tests and exams.	Mental strategy, activating the personal higher order goal of wanting to outperform others, activating social reference norms to compare achievement Secondary control	Low task value, enhancing personal significance and utility value	•Internalized achievement standard, using social comparisons •Performance-approach goal orientation • Cooper and Corpus (2009): 8 year-olds mention this strategy frequently nearly to the same amount as older learners, including adults, but neither age group gave adequate reasons why this strategy is useful. → 8–10 years?
Performance-avoidance self-talk	Engelschalk et al. (2015); Schwinger et al. (2007)	I imagine how it would feel to do worse than my fellow students.	Mental strategy, activating the personal higher order goal of avoiding to do worse than others, activating social reference norms to compare achievement Secondary control	Low task value, enhancing personal significance and utility value, side effects: potentially enhances emotional costs and decreases wellbeing	•As above
Highlighting personal goals	Engelschalk et al. (2015); Schwinger et al. (2007); Wolters (1998, 1999)	I remind myself of the goals I pursue with my learning.	Mental strategy, enhancing the salience of personal goals to highlight the positive consequences of the action Secondary control	Low task value, enhancing utility and identified/integrated motivation	•Representation of higher order personal goals, goal hierarchy, concept of utility value for these goals, feelings of purposelessness and indifference •May also depend on the type of higher order goal (e.g., a rather short-term goal such as obtaining a reward or a very long term occupational goal such as becoming a doctor) → 10–18 years?
Enhancement of personal significance	Miele and Scholer (2018); Schwinger et al. (2007); Wolters (1998, 1999)	I try to link what I am supposed to learn with my own experiences.	Mental strategy, altering appraisals of the task or content to relate to personally important aspects Secondary control	Low subjective task value, enhancing personal significance of the task or task content	•Self-concept/identity to assess attainment value of a task and construct reasons for attainment value •Feelings of indifference and purposelessness •10–14?
Reprioritizing	Hofer and Fries (2016)	If I would like to do something other than study, I tell myself that the other thing can wait.	Mental strategy, shifting goals in the goal hierarchy, making a desired action alternative less significant in comparison to a target goal or vice versa Secondary control	Solving a motivational action conflict, reducing task value of action alternative or increasing the value of a cognitively preferred alternative	•Explicit mental representations of a goal hierarchy •Concepts of task utility or personal value for short term or higher order goals → 8–10 years?
Multifinal actions	Hofer and Fries (2016); Miele and Scholer (2018)	Choosing actions which unify two goals and needs to satisfy both simultaneously.	Solving a motivational action conflict, the achievement of two goals that are pursued simultaneously no longer have to be mutually exclusive, but are pursued through one action. primary control	Reducing opportunity costs	•Simultaneous representation of two equally important personal goals; •Feelings of temptation, conflict between them, distraction or regret about not doing one (opportunity costs) •Knowledge about equi- and multifinality of actions •Late adolescence?

Arrows followed by age refer to the approximate age at which this control strategy is expected to start occurring.

## 2 Theoretical models describing motivational self-regulation

**Figure 1:**
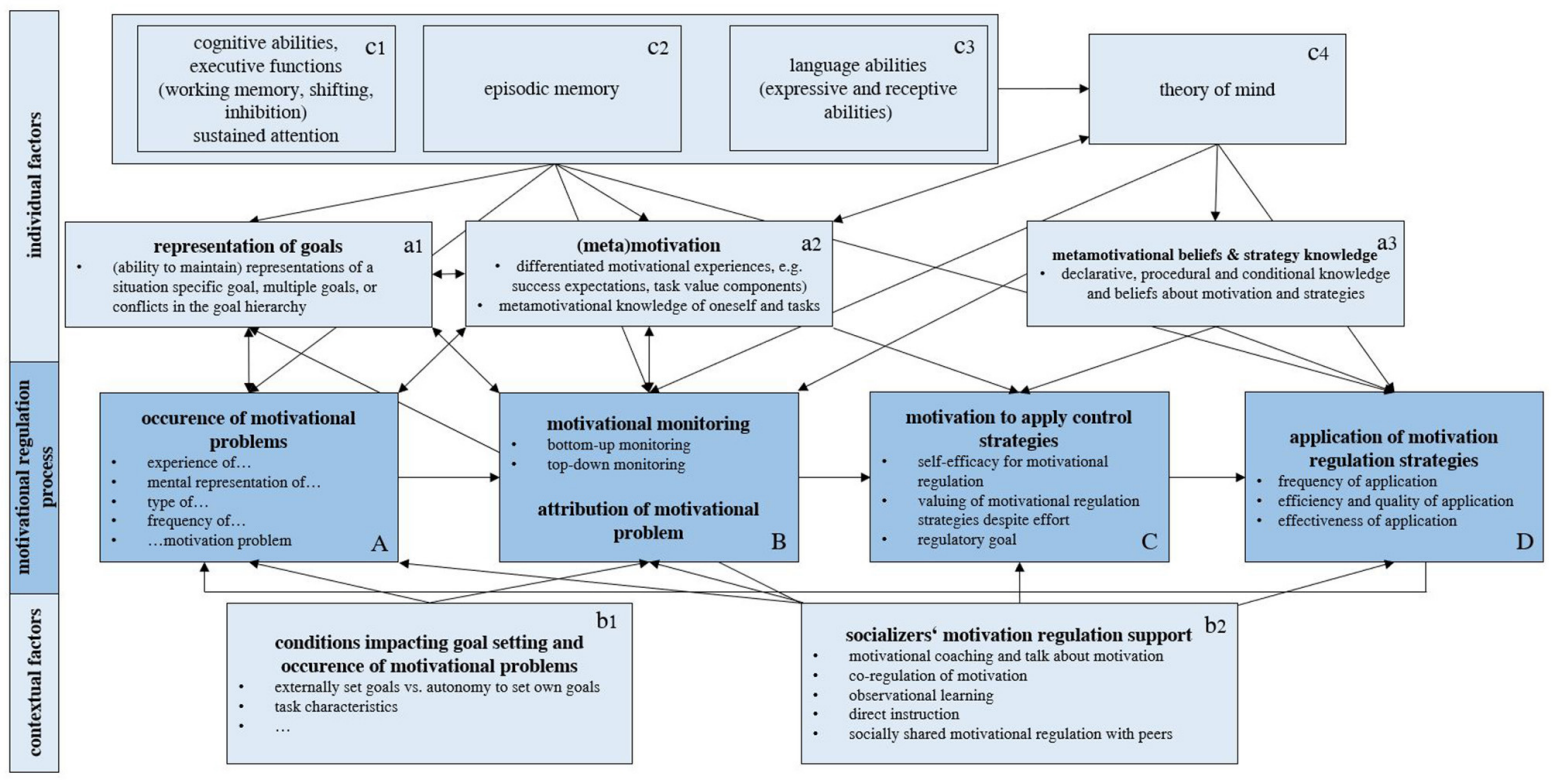
Conceptual framework for investigating the development of motivation regulation.

To date, two models explicitly describe motivational self-regulation at a situational level: The process model of motivation regulation (Table 1, column A; Schwinger and Stiensmeier-Pelster, 2012) and the metamotivational model of motivation regulation (Table 1, column B; Miele and Scholer, 2018; Miele et al., 2024). However, neither model makes explicit reference to ontogenetic aspects. In contrast, the motivational theory of life span development (Table 1, column C; Heckhausen et al., 2010; Heckhausen and Schulz, 1995) suggests longitudinal changes of motivation regulation beyond academic contexts. Although we acknowledge that other theories may have presented similar ideas in the past (e.g., the concept of psychological defenses from psychoanalytic theories, Cramer, 2015) or current frameworks including concepts overlapping with processes of motivational self-regulation (such as the extant literature on general coping, Skinner and Zimmer-Gembeck, 2007), in the current review, we focus on theories more narrowly focused on motivational self-regulation. These models agree that motivation regulation is a process in which several aspects build on each other (Figure 1, dark blue boxes). This process starts with the occurrence of a motivational problem (Figure 1, box A). The next key aspect of the self-regulatory process is to monitor this problem (box B), which includes noticing discrepancies between one's current motivational experience and a desired state. The monitoring process also includes attributing the problem to a cause and to develop an understanding of the direction in which motivational experiences should be changed. Finally, based on the results of this monitoring process, strategies to regulate one's own motivation can be selected and applied to remedy the respective motivational problem (box D). An important precondition here is whether one is motivated to fix this state (box C). Many models of self-regulation propose that this is a circular process in which the application and results of motivation regulation strategies are monitored, resulting in yet other or more attempts to regulate motivation (e.g., Carver and Scheier, 1990; Zimmerman, 2002). The key propositions of each theory regarding core aspects of motivation regulation and its development are discussed in the following. Table 1 provides an overview of helpful articles on the models and frameworks (Table 1, header), the roots and origins (Table 1, row 1), and main implications of each theoretical strand to facilitate comparisons between them (Table 1, rows 2–9).

### 2.1 Occurrence, monitoring, and attribution of a motivational problem

The starting point of situational motivation regulation is the occurrence and perception of a motivational problem (Figure 1, box A). Both the process model of motivation regulation (Schwinger and Stiensmeier-Pelster, 2012) and the metamotivational model of motivation regulation (Miele et al., 2024; Miele and Scholer, 2018) conceptualize the perception of low motivation for a task or misfit of perceived quality of motivation to task demands as the starting point of the motivation regulation process (Table 1, row 2). According to the motivational theory of life span development, people strive for agency through primary control from an early age by setting their own goals and adapting the environment to their needs (Heckhausen et al., 2010; Table 1, row 2). Motivational problems thus occur whenever primary control opportunities of the environment or individual capacities limit primary control striving. This implies two developmental prerequisites: Children need a representation of an action goal, a desire or need that they want to pursue or fulfill, and they must be able to maintain it if disruptions occur on the way to goal attainment, for example, in the form of impulses for an alternative action or conflicts between action goals (Figure 1, box a1).

“Motivational monitoring”, recognizing motivational problems (Figure 1, box B; Table 1, row 3), may involve both explicit, top-down monitoring, during which one actively examines current actions for motivational problems, and bottom-up monitoring, which becomes apparent through interfering metamotivational feelings. Metamotivational feelings refer to emotions that can indicate specific motivational problems, for example, boredom for low intrinsic value, or anxiety for low success expectancies. Thus, children have to be able to realize that they are not sufficiently motivated (quantity) or not in the right way (quality) to start or continue a task.

Next, Schwinger and Stiensmeier-Pelster (2012) propose that people must experience a need for higher motivation in order to change something about low motivation. This need may result from a superordinate goal, personal values attached to a task or goal, ought/should-considerations, societal norms and expectations, and expected external rewards and punishments (Heckhausen et al., 2010; Miele and Scholer, 2018; Schwinger and Stiensmeier-Pelster, 2012, cf. Table 1, rows 2 and 4). Without this, one could simply quit the task without taking measures to change things. Motivation to self-regulate one's motivation may thus depend on how strongly children value the task or goal they would like to achieve and whether regulating one's motivation seems worth the effort (Perry et al., 2019), but also on beliefs regarding whether motivation is malleable through personal effort or fixed (Figure 1, box C; O'Keefe et al., 2018; Thoman et al., 2020; Trautner and Schwinger, 2022). This extends the first prerequisite beyond relatively short-term goal representations: Children additionally need higher order personal goals or values attached to a goal, or at least an anticipation of external rewards or punishments, to also experience the need for higher motivation.

As a final aspect of the monitoring part of motivation regulation, people search for the cause of the motivation problem (Figure 1, box B; Table 1, row C); for example, whether the problem stems from task difficulty, insufficient task value (cf. Engelschalk et al., 2015; Wigfield et al., 2015), or a mismatch between the quality of one's motivation and task demands (Miele and Scholer, 2018). This attribution can be implicit, for example through the quality of metamotivational feelings accompanying motivational problems, indicating specific types and causes of motivational problems (Miele et al., 2024; Miele and Scholer, 2018). It can also be more explicit, depending on individual knowledge and beliefs regarding one's own motivation (metamotivational self-knowledge), motivational incentive structures of the environment and the task (metamotivational task knowledge) and how they interact (Figure 1, box a2; Fujita et al., 2024; Miele et al., 2024). The motivational theory of lifespan development does not explicitly highlight attributions to motivational problems, but suggests that regulation strategies are selected upon specific problems, implying some degree of monitoring and attribution (Heckhausen et al., 2010; Table 1, cell C3). Empirically, Gurland and Glowacky (2011) demonstrated that eight-to-twelve-year-olds differ in beliefs about which motivational task characteristics (e.g., rewards, punishments, choices) affect different types of tasks (short-term vs. long-term, exciting or boring) and in which ways they do so. These findings suggest that children's knowledge and ideas about their own motivation, tasks, and incentives can shape their perception of motivational problems and approaches to regulation. For the development of motivation regulation, this poses the question not only of whether, but which motivational problems children encounter and how metamotivational task and self-knowledge develop. For example, children may experience motivational difficulties due to low interest or intrinsic motivation very early on, as interest becomes channeled at specific objects or when school demands do not match their interests, resulting in decreasing joy (e.g., Anderson and Perone, 2024; Lichtenfeld et al., 2023). However, to experience motivational difficulties due to low personal attainment value, one may need a more elaborate representation of personal values. Attainment value may mean rather different things to children of different ages (Wigfield et al., 2015). Some personal values may emerge early and refer to broad descriptions, such as wanting to be a good child (Harter, 2012), while others, such as attributions regarding the causes of these problems and whether they are correct, may develop somewhat later. This prompts the question of how different motivational problems emerge and to what extent children encounter them. It also includes whether they are able to form stable mental representations and knowledge about self- and task motivation, make appropriate attributions of causes for motivational problems, and which conclusions they draw from this for regulation.

### 2.2 Motivational control via motivation regulation strategies

To change one's own motivation, motivation regulation strategies are used depending on the causes of the respective motivation problem (Figure 1, box D; Table 1, row 5). Wolters (2003) defines this as “activities through which individuals purposefully act to initiate, maintain, or supplement their willingness to start, to provide work toward, or to complete a particular activity or goal (i.e., their level of motivation). This form of regulation is achieved by deliberately intervening in, managing, or controlling one of the underlying processes that determine this willingness (i.e., the processes of motivation). Regarding behavior, the regulation of motivation encompasses those thoughts, actions, or behaviors through which students act to influence their choice, effort, or persistence for academic tasks” (p. 190). These strategies include concrete behavioral strategies such as environmental control and self-consequating (cf. Cooper and Corpus, 2009) or increasing social integration, as well as an even broader spectrum of mental strategies, such as emphasizing the salience of cognitively preferred goals or higher-order goals (mastery, performance-approach and -avoidance self-talk, reprioritization, deprioritisation), strategies that explicitly increase task value (enhancement of situational interest, personal significance) or change situational expectations of success (self-efficacy self-talk, proximal goal setting). Remembering introjected reasons for motivated behavior could also be one of these strategies (Miele and Scholer, 2018; Schwinger et al., 2007). A very advanced strategy for regulating motivational action conflicts refers to selecting multifinal actions (Hofer and Fries, 2016; Miele and Scholer, 2018). In addition, qualitative studies with learners have also reported potentially maladaptive strategies, such as multitasking (Hofer and Fries, 2016), lowering standards to achieve goals (Hofer and Fries, 2016; Schwinger et al., 2007), and procrastination to seek pressure (Engelschalk et al., 2015; Hofer and Fries, 2016; Schwinger et al., 2007; see Table 2 for specific strategies for motivation regulation discussed in previous works across the theoretical strands displayed here, including examples and sample references on the theoretical and empirical background of these strategies). The motivational theory of life span development differentiates between primary control, where individuals modify the environment to meet their needs, and secondary control, where strategies are aimed at changing the self—one's own actions, thoughts, and preferences—to match environmental demands (Table 1, cell C5; Heckhausen et al., 2010). Most of the mentioned strategies are secondary control strategies as they aim at adapting one's beliefs and attitudes to the environment, higher order, or externally set goals. Only a few strategies explicitly target the environment (e.g., environmental control). Additionally, the motivational theory of life span development suggests some secondary control strategies that do not necessarily fall under the definition of motivation regulation strategies as defined by Wolters (2003); for example, because they are not used intentionally with the aim of enhancing motivation (e.g., reattributing an incident of failure without explicitly doing it to enhance motivation).

The use of motivation regulation strategies implies that children know these strategies (declarative strategy knowledge), know how to implement them (procedural strategy knowledge) and use them appropriately for a problem (conditional strategy knowledge, Figure 1, box a3). Beyond knowledge, both cognitive and, in part, motor skills, as well as the willingness to use the strategies are required to effectively implement strategies (Spörer and Brunstein, 2006). Due to the different motivational processes strategies aim at (e.g., changes in evaluations of task value or expectations of success, manipulation of environmental conditions, changes in the evaluation of goals such as reprioritization, see Table 2), it can also be assumed that the knowledge and application of strategies develop depending on the cognitive, motivational-emotional, linguistic and motor prerequisites required for each strategy and motivational problem. Thus, these prerequisites may vary by strategy and will not develop simultaneously. The motivational theory of life span development also suggests that the capacity for secondary control is not innate, but acquired—a process starting during childhood and continuing into old age (Table 1, cell C8).

### 2.3 Individual factors influencing the motivation regulation process

All theories recognize various individual factors influencing the motivation regulation process (Table 1, row 6). These include the aforementioned individual beliefs about one's own motivation and metamotivational knowledge (Miele and Scholer, 2018; Schwinger and Stiensmeier-Pelster, 2012; Trautner and Schwinger, 2020, 2022), as well as strategy-specific cognitive and motivational-emotional prerequisites (Figure 1, boxes a1-3). In addition, Schwinger and Stiensmeier-Pelster (2012) cite personal characteristics such as personality traits as influencing factors. This is in line with the broad perspective that many individual factors can support or impede primary and secondary control capacities (Heckhausen et al., 2010; Heckhausen and Schulz, 1995). These characteristics may not only impact the motivation regulation process itself, but also its development. Comparable studies suggest that early childhood temperament influences emotional development and emotion regulation (Lohaus and Vierhaus, 2019).

### 2.4 Contextual factors influencing the motivation regulation process

In addition to individual factors, all theories more or less explicitly mention the influence of contextual factors (Table 1, row 7). They differ, however, in which factors are mentioned. For example, motivation regulation may vary by task characteristics (highly vs. less-structured tasks) or the setting (e.g., in sport, academic tasks, etc., Schwinger and Stiensmeier-Pelster, 2012), although this has rarely been studied (Trautner et al., 2025). This is mainly in line with propositions from the metamotivational model of motivation regulation, which suggests that motivational task demands and characteristics afford specific types of motivation and influence how motivation is regulated (Miele et al., 2024; Miele and Scholer, 2018). The motivational theory of life span development takes an even broader stance on how context influences the motivation regulation process, suggesting that environmental characteristics can provide opportunities and constraints to individuals' primary and secondary control, for example, via direct physical constraints, but also societal norms (Heckhausen et al., 2010; Heckhausen and Schulz, 1995). It is conceivable that certain types of contexts that children experience may bring with them new motivational problems (comparable, for example, with the concept of stage-environment fit or misfit by Eccles and Roeser, 2011 or environments limiting individuals' opportunities for primary control according to Heckhausen et al., 2010 Figure 1, box b1) and thus provide an opportunity for or limitation of the development of motivation regulation. Neither Miele and Scholer (2018) nor Schwinger and Stiensmeier-Pelster (2012) make any statements in their models about social factors influencing motivation regulation, for example the role of external regulation of one's motivation by others. However, previous research has shown that motivation regulation not only occurs at the individual level, but also in dyadic and group contexts (co-regulation and socially shared regulation of motivation, e.g., Panadero and Järvelä, 2015). Emotion regulation is explicitly described as a process that is initially strongly characterized by interpersonal regulation (or co-regulation) by caregivers, but gradually shifts to intrapersonal self-regulation (Holodynski, 2006). As emotions and motivation are conceptually overlapping constructs, it can be assumed that a similar developmental trend could also be seen with regard to motivation regulation (Figure 1, box b2; cf. Stockinger et al., 2025).

### 2.5 Outcomes of the motivation regulation process

When successfully applied, motivation regulation strategies change motivation (Miele and Scholer, 2018; Schwinger and Stiensmeier-Pelster, 2012). Motivation is not a unitary construct and includes many of the different motivational experiences mentioned above, such as interest, task value, intrinsic and extrinsic aspects of motivation, goal adjustment, and success expectancies (Heckhausen et al., 2010; Miele and Scholer, 2018; Ryan and Deci, 2000; Wigfield et al., 2015). Such motivational experiences are the most direct outcomes of the motivation regulation process (Heckhausen et al., 2010; Miele and Scholer, 2018), and in turn result in motivated behaviors (Schwinger and Stiensmeier-Pelster, 2012). At this behavioral level, self-reported willingness to exert effort is the most frequently examined outcome of the motivation regulation process (Trautner et al., 2025). Strictly speaking, this is a behavioral intention, but not actual behavior. Therefore, especially when examining effort as an outcomes of the motivation regulation process, (different operationalizations of) time on task, such as the time actually spent on a task may be an appropriate indicator of behavioral aspects of motivation (Godwin et al., 2021). Terms also used to describe such behavioral aspects of motivation further include (task) persistence as one's continued efforts toward a goal over a longer period of time, even when facing difficulties and obstacles (Ishikawa and Kanakogi, 2025; Oeri and Roebers, 2021), and endurance, which more strongly refers to physical activity and muscular abilities to continue effort (e.g., Obert et al., 2003).

Among the more distal and long-term outcomes of the motivation regulation process are achievement (for example, academic achievement, goal attainment), and wellbeing (Heckhausen et al., 2010; Miele et al., 2024; Schwinger and Stiensmeier-Pelster, 2012). However, most evidence showing these positive associations between motivation regulation strategy use and achievement, effort, or wellbeing comes from cross-sectional studies (Fong et al., 2024; Trautner et al., 2025). Thus, little is known about how early motivation regulation is related to later motivation regulation and respective positive outcomes. However, drawing on the relevance of early self-regulation on later self-regulation, wellbeing, and success in life (for a meta-analytic review, see Robson et al., 2020; regarding emotion regulation: Klein et al., 2022; coping: Zimmer-Gembeck and Skinner, 2011; executive functions: Yang et al., 2022; self-control: Mischel et al., 1988; Watts et al., 2018), it can be argued that early motivational self-regulation may also be important for later outcomes. Therefore, examining how motivation regulation develops and how early motivation regulation is related to later motivation regulation, academic and personal success, and wellbeing later in life is important. As the motivation regulation process contains several aspects, developmental preconditions for all of these need investigation.

### 2.6 Summary: prerequisites for motivational self-regulation

In summary, developmental preconditions for effective motivation regulation are (a) that (different types of) motivational problems occur, (b) that one is able to monitor and notice this problem, (c) that one attributes it to a motivational cause, (d) that one has a representation of a higher order goal or incentive to be motivated to take measures to eliminate this motivational problem, and (e) that one has the means to effectively regulate this motivational problem (e.g., in the form of declarative, procedural, and conditional strategy knowledge and the cognitive, motivational-emotional, motor, and linguistic prerequisites to execute these strategies successfully). It can also be assumed that these prerequisites are influenced by various individual, contextual and social factors.

## 3 Development of components of motivational self-regulation

These components outlined by theoretical accounts on motivational self-regulation may develop differently and interdependently, depending on several preconditions or precursor abilities. There is some research on motivation regulation in pre- and primary school children showing that they already (know how to) regulate their motivation and that several factors impact self-regulatory abilities (e.g., Cooper and Corpus, 2009; Dörrenbächer and Perels, 2018; Ebbes et al., 2024; Grüneisen et al., 2024; Kuhl and Kraska, 1989; Nader-Grosbois, 2014). However, these studies differ in the theoretical conceptualization and operationalizations of motivation regulation, the age group, and the (educational) setting assessed, as well as the components of the motivational regulation process under investigation. The following section summarizes how different aspects of the motivation regulation process may develop.

### 3.1 Representation of goals and maintenance of goal representations

Goals, represented in Figure 1, box a1, can be defined as hierarchical knowledge structures regarding “desirable future state of affairs” (Shah and Kruglanski, 2000, p. 85). Goals energize actions as they represent an “intention to accomplish a task, to achieve some specific state of the world or take some mental or physical action” (Altmann and Trafton, 2002, p. 39). Goal-directed behavior can be observed in young children as, during their second year of life, their actions increasingly shift from being conducted due to the action itself toward anticipated effects and results of an action (Heckhausen and Heckhausen, 2018). The strength and accessibility of goal representations develop during preschool years and become increasingly associated with goal-directed behavior (Munakata, 2001). Even when facing two conflicting action options, first-graders already have reliable cognitive-semantic representations of their goals (Kuhl and Kraska, 1989). To maintain goal representations, two core executive functions (Diamond, 2013) play an important role: working memory capacity and updating are important to hold one's goal in mind, while inhibition is relevant to shield it from distractions, and both depend on working memory capacities (Figure 1, box c1).

### 3.2 Higher order goals or values, understanding conflicting goals, and motivation to self-regulate

A lack of motivation by itself or the experience of motivational conflict is not sufficient for further motivation regulation—a need for higher motivation or conflict resolution is necessary (Schwinger and Stiensmeier-Pelster, 2012). This need for higher (or qualitatively different) motivation may result from the anticipation of externally set rewards and punishments for not doing an activity or from representations of a higher order personal goal (Heckhausen et al., 2010; Miele and Scholer, 2018; Pintrich and Zusho, 2002; Schwinger and Stiensmeier-Pelster, 2012). Similarly, in the domain of volitional control, Kuhl and Kraska (1989) proposed that to exert volitional control, children need a self-selected or self-consistent goal preference for a different, less emotionally charged alternative action, even if this conflicts with a current emotionally charged action impulse. “Self-selected” may be interpreted broadly in the face of anticipation of external rewards/punishments as children can decide whether they seek to obtain or avoid these despite incentives being externally administered. This implies that one must be able to remember such a higher order goal and the reasons for pursuing this goal. Research on the role of goal hierarchies in action planning and control shows that 3-year-olds still have difficulties keeping goal hierarchies in mind and shifting between lower-order and higher-order goals, but that there are substantial improvements in this area during the preschool period (e.g., Freier et al., 2017; Yanaoka and Saito, 2017). These improvements are related to the development of different components of executive functions (Freier et al., 2017; Miyake and Friedman, 2012), but also in episodic memory needed to envision past and future desired states (Figure 1, box c2; Holodynski et al., 2013). Again, during kindergarten and into preschool years, significant improvements occur in these abilities (Diamond, 2013).

Another developmental trend of importance here may be that during childhood and adolescence (and most likely across the lifespan), goals and hierarchies emerge and change with respect to their content depending on personality, environmental factors, and social influences (Heckhausen et al., 2010; Heckhausen and Schulz, 1995). Thus, besides interindividual differences in cognitive prerequisites for maintaining goal hierarchies in childhood (Diamond, 2013; Jacob et al., 2021), there may be many important shifts in goal content and quality (in terms of self-congruence) early on as children develop in terms of their personality and social abilities, depending on children's knowledge about themselves and their own preferences in the sense of “motivational competence” (Grund et al., 2018; Kuhl and Kraska, 1989; Rheinberg and Engeser, 2012). In turn, setting many self-congruent goals (as a means of primary control) may reduce the need to regulate one's motivation in terms of secondary control (Heckhausen et al., 2010), implying that early motivational goal congruence may lead to a later acquisition of motivational control. This poses the question of how actively children are able and willing to set these goals for themselves, matching their temperament/personality or whether environmental factors prevent them from selecting self-congruent goals in the sense of an early stage-environment misfit regarding autonomy (Eccles and Roeser, 2011). Similarly, Pintrich and Zusho (2002) suggest that acquiring self-regulation skills depends less on age or age-related maturation, but on the active acquisition of self-regulation depending on experiences one makes, for example, motivational problems one encounters.

### 3.3 Developmental aspects of the emergence of different motivational problems

As children grow older, both the overall frequency of motivational problems and the type of motivational problem may change. Regarding the frequency of encountering problems, children may increasingly face more externally (and not yet self-congruent) goals as they grow older because their environments become less adaptive to their personal needs in the sense of a decreasing stage-environment fit (Figure 1, box b1; cf. Eccles and Roeser, 2011; Heckhausen et al., 2010). For example, during primary school, children's ability to use social comparisons to infer their own ability increases, which does not always lead to favorable self-evaluations (Harter, 2012), and they are expected to fit into larger groups. Simultaneously, they develop abilities to more autonomously set their own goals, not necessarily in accordance with what their environment expects of them. Developmental trends of declining intrinsic motivation or interest and domain-specific ability self-concepts during school years have been reported consistently, although there is considerable heterogeneity in developmental trajectories (e.g., Cole et al., 2001; Gaspard et al., 2020; Jacobs et al., 2002; Orth et al., 2021; Wigfield et al., 2015). For the occurrence of motivational problems, this implies that children may encounter motivational problems increasingly often in specific domains. Simultaneously, younger children may receive more (individualized) support from caregivers when facing a motivational problem, for example, through caregivers creating motivationally engaging environments to prevent motivational conflicts or co-regulating children's motivational problems (Holodynski et al., 2013), while older children receive less support and are expected to self-regulate. This may create more opportunities requiring self-regulation (Wolters and Pintrich, 1998; Zimmerman, 2013).

Overall, although from their early weeks on, children explore their environment with curiosity and intrinsic interest, they may already experience motivational conflicts between two attractive but mutually exclusive action goals which cannot be enacted at the same time (Hofer and Fries, 2016) as soon as they are cognitively able to hold two competing action goals in mind. In addition to changes in the frequency of motivational problems, the type or quality of problems may change as interests, valued actions, and (ability) self-concepts become more differentiated over time.

### 3.4 Development of monitoring and attribution to motivational problems

Regarding the monitoring of motivational problems, bottom-up and top-down processes have been proposed (Miele and Scholer, 2018; Schwinger and Stiensmeier-Pelster, 2012). During bottom-up-monitoring, so called “metamotivational feelings” indicate the occurrence of specific motivational problems (Miele and Scholer, 2018). For example, boredom signals low intrinsic value, interest, and understimulation during or in anticipation of a task, while anxiety signals low success expectancies. However, not all these emotions may be present from birth but evolve from more general feelings of discontentment. General discontentment, indicating an absence of intrinsic value or understimulation, may occur within the first 2 years of life (Holodynski, 2006). Boredom, purposelessness, or indifference, indicating low intrinsic or self-relevant value, however, may evolve much later. For example, boredom, indicating low interest, seems rarely verbalized by or observed in 2- to 4-year-old children themselves (Wellman et al., 1995) but it is observable in 4- to 6-year-olds (Anderson and Perone, 2024), and reported by primary school children (e.g., Camacho-Morles et al., 2021; Lichtenfeld et al., 2023). Anxiety, frustration, and hopelessness, signaling low self-efficacy, may also be experienced at this age (Camacho-Morles et al., 2021; Harter, 2012). However, the question remains whether children at that age also have the correct mental representation of the source of their motivational experience. For example, preschool and early school children have difficulties disentangling effort from (lack of) ability (Folmer et al., 2008). Also, it is not clear at which age children distinguish between different aspects of task value (e.g., have representations of attainment or utility value beyond intrinsic value). Thus, it may not be possible for them to differentiate and attribute motivational problems due to effort costs or low success expectancies as this depends on differentiated concepts of the two.

Since children's ability to verbalize emotions and understand their causes develops a little later than the mere experience of those emotions (e.g., Pons et al., 2004), it can be expected that top-down monitoring processes and attributions of motivational problems to causes may evolve subsequently. Top-down monitoring may evolve somewhat later during preschool and early school years, as research on metacognitive control (one's ability to monitor and control one's thoughts; Nelson and Narens, 1990; Winne and Perry, 2000) shows. Metacognitive monitoring seems to evolve earlier than metacognitive control (e.g., Bryce and Whitebread, 2012). For both monitoring and control, this observational study revealed differences in the frequency and type of strategies used between 5- and 7-year-old children. This indicates that self-regulation develops in response to a presenting problem and thus occurs somewhat later in the course of development than the perception of problems in the execution of actions. Research on motivational monitoring in pre- and primary school children to date mostly uses observational tools and has found that children make utterances about their motivational states, indicating some awareness about them, and that there are some improvements regarding the frequency of monitoring (e.g., Grau and Whitebread, 2012; Zachariou and Whitebread, 2019). However, these studies do not differentiate between top-down and bottom-up monitoring. Important correlates and developmental preconditions frequently mentioned in research on metacognition are the development of executive and general cognitive functions (Diamond, 2013) and the development of theory of mind, as distinguishing one's own beliefs, assumptions, and goals from those of others indicates that children can reflect on their own thinking.

### 3.5 Development of motivational control

The development of motivational control includes acquiring declarative, procedural, and conditional knowledge about thoughts, behaviors, and strategies aimed at enhancing different aspects of one's motivation depending on one's motivational problem (Figure 1, box a3; Spörer and Brunstein, 2006; Wolters, 2003). This implies that different motivation regulation strategies may develop in correspondence with the occurrence of motivational problems, as well as the ability to monitor and attribute motivational problems. Thus, not all strategies will develop simultaneously. However, observing motivational problems in others may be sufficient for children to produce adequate, spontaneous solutions to these problems (Cooper and Corpus, 2009). Studies investigating knowledge and use of motivational strategies indicate that pre- and primary school children know and use several strategies (e.g., Cooper and Corpus, 2009; Dörrenbächer and Perels, 2018; Grüneisen et al., 2024); however, studies often combine several strategies into one motivation regulation index and rarely assess both monitoring and control, making it difficult to draw conclusions about the extent to which some behaviors or thoughts are explicitly used to regulate motivation.

Previous research on motivation regulation has described several strategies to regulate one's motivation. These strategies vary in the motivational target they seek to change and in developmental preconditions required for their effectiveness. Table 2 summarizes these strategies, their theoretical and empirical background (including sample references for further reading), their targets and means, and their hypothesized developmental preconditions, including a tentative estimation of an age range in which children are likely to acquire these strategies.

Besides motivational problems as a “cause of necessity” to develop specific motivation regulation strategies, the demands of specific strategies may influence when and how they are acquired. Behavioral, concrete strategies, such as environmental control or leaving a situation, have been found to be present at an earlier age compared to more mental, abstract, or cognitively more demanding strategies (Cooper and Corpus, 2009; Holodynski, 2006). These strategies may also be more easily acquired through observation compared to complex intrapsychic strategies. Thus, cognitive, motor, language, or other prerequisites may play a role in the acquisition of specific strategies.

When developing strategies to regulate motivation, the question arises whether some strategies are more or less adaptive or developmentally appropriate (Table 1, row 9). Previous literature on motivation regulation strategies in mainly adult samples has mentioned that some strategies seem to have costs (e.g., for wellbeing, emotional costs, or else, e.g., Schwinger and Otterpohl, 2017). For example, performance avoidance self-talk (reminding oneself to avoid performing worse than others, Table 2) may on one hand increase extrinsic motivation to continue studying. On the other hand, it may be detrimental for affective wellbeing if one constantly reminds oneself of being outperformed by others. For most strategies examined to date, however, the focus was less on whether they are adaptive or not in general, but on whether they are applied in suitable situations to suitable goals, and are not merely at the service of short-term, but also long-term goals (Heckhausen et al., 2010; Miele and Scholer, 2018; Schwinger and Stiensmeier-Pelster, 2012; von der Mülbe et al., 2024). This implies that a strategy can be adaptive and useful for increasing motivation and goal achievement despite negative side effects on other outcomes. In the case of performance avoidance self-talk, its use could be adaptive if the person believes that she can master the task, but should get going with it. If this were not the case, if she believed she might not be able to master the task and the motivational problem at hand was due to low success expectancies as opposed to low task value, the strategy may come with more costs than benefits and may be maladaptive. Regarding adaptivity, the motivational theory of life span development suggests that primary control strategies have “functional primacy” over secondary control strategies, meaning that there is larger value in adapting the environment to one's needs to foster individual growth and development (Table 1, cell C9). This implies that at younger ages, when individuals' abilities to execute primary control striving grow and the environment offers multiple opportunities for primary control, it would be more adaptive to develop primary control strategies first. This does not mean that primary control is always adaptive and secondary control is not—for one, secondary control is adaptive for the individual when primary control is not feasible due to limited personal capabilities and resources or when environmental constraints prevent this. Second, primary control is regarded as dysfunctional when it is not based on valid estimations of contingencies between actions and outcomes in the real world (Heckhausen and Schulz, 1995). Additionally, a clear categorization of strategies as either primary or secondary control is not always possible. Although some strategies have unequivocally been labeled as dysfunctional (e.g., self-handicapping, Heckhausen and Schulz, 1995), more research on short- and long-term consequences of strategies both on motivational, but also on additional outcomes is needed to investigate adaptiveness and age appropriateness.

### 3.6 Development of motivation to apply motivation regulation strategies

Being able to hold an attractive higher order goal in mind alone may not suffice to regulate one's motivation; being motivated to apply control strategies as a means to reducing the discrepancy between experienced and desired motivation may be necessary. Motivation to apply strategies encompasses a diverse set of beliefs about (a) whether or not one can change motivational experiences and (b) whether one wants to do so, given that applying strategies is an effortful activity (Pintrich and Zusho, 2002). Regarding the first set of beliefs, theories about whether motivational experiences generally can be changed at all (malleability beliefs about motivation; O'Keefe et al., 2018; Thoman et al., 2020; Trautner and Schwinger, 2022), and self-efficacy beliefs about motivational self-regulation, such as whether one is able to change one's motivation in the face of boring or difficult tasks (Trautner and Schwinger, 2020, 2022), are related to the likelihood of applying motivation regulation strategies. Additionally, lower secondary school students reported higher self-efficacy and value for applying self-regulated learning strategies in general when their teachers also reported using these strategies and correspondingly promoted self-regulated learning in the classroom (Jud et al., 2024). Regarding seeing value in applying strategies, beliefs about the effectiveness of specific strategies and whether they are worth the effort may be important predictors of whether or not children choose to regulate (Pintrich and Zusho, 2002). However, whether and which beliefs (preschool) children hold about the motivation regulation process has received little attention to date.

## 4 How do children acquire motivation regulation skills?

Several mechanisms have been suggested regarding how children acquire motivation regulation. Most prominently, learning in interactions from caregivers or other role models is mentioned (Figure 1, box b2). Miele et al. (2024) additionally suggested logical reasoning and trial and error experimentation may serve as individual learning mechanisms that form people's insights into their motivational experiences and ways to regulate them. This may be especially important in forming metamotivational representations (i.e., knowledge about one's own motivation and motivational structures of tasks) as situational experiences of motivation, motivational problems, and attempts to deal with them accumulate over time to form more stable representations (Skinner and Zimmer-Gembeck, 2007). However, these mechanisms have not yet been tested with regard to motivation regulation in children and adolescents.

### 4.1 Direct instruction and observational learning

Motivational self-regulation is often conceptualized as one domain under the umbrella of self-regulated learning (Pintrich, 2004; Zimmerman, 2002; Trautner et al., 2025). Regarding the acquisition of self-regulated learning strategies in general, Zimmerman (2013) proposed an observational learning framework. In a first step, learners observe successful models applying strategies. These strategies are increasingly used autonomously, e.g., independent from social and external reinforcement, until they are applied flexibly and dynamically during learning. Accordingly, competent models applying these strategies may play a decisive role by pointing out relevant aspects of strategy application in critical phases for the acquisition of motivation regulation strategies, and providing increasing independence in strategy use (Miele et al., 2024; Thompson, 1991). Direct strategy instruction by parents or teachers, such as suggesting actions and thoughts to enhance one's motivation or persist at a task, may be imitated and become internalized over time (Morris et al., 2007; Thompson, 1991). This may also occur implicitly, while children observe others experiencing and dealing with motivational problems, independently of successful outcomes in such situations. Therefore, socializers' motivation regulation abilities may influence children's acquisition implicitly and in non-intended ways (Morris et al., 2007).

### 4.2 Parenting practices supporting (motivational) self-regulation

Several theories highlight the importance of caregivers' behaviors for the development of self-regulation. Early theorizing on how children acquire knowledge about their inner and outer world and make sense of perceptual input proposes that internalization of language and patterns of behavior are central (Vygotsky, 1978). Such internalization processes are heavily guided by language, but also by caregivers' scaffolding. This idea is not only important because it emphasizes gradual development and acquisition of concepts and skills, but also because it can serve to explain interindividual variability in skills, depending on larger interpersonal, societal, and cultural contexts (Miller, 2022). Similarly, sociocultural and constructivist approaches to the development of emotions, emotional self-regulation, and coping highlight that both the meaning of emotions and their regulation are formed and learned in social interactions (Holodynski, 2006; Skinner and Zimmer-Gembeck, 2007). Central to this understanding is that during the early years, children rely on caregivers to fulfill their needs, for which emotions have a communicative function. Due to this scaffolding, children learn to name emotions, stressors, and their causes, and to take control of fulfilling the respective underlying needs themselves over time. This depends on improving cognitive and motor abilities enabling self-regulation, but also on a more explicit understanding of the functions and causes of different emotions, and ways to regulate them independently from caregivers. To this end, specific parenting practices such as emotion coaching and co-regulation are important (Silkenbeumer et al., 2024). Emotion coaching includes parenting practices focusing on responsively attending to a child's emotions to increase emotional awareness, for example, by observing and labeling a child's expressed emotion, mirroring and validating it, and conversations about emotions with the child. Co-regulation of emotions goes beyond increasing emotional awareness by supporting emotion regulation, for example, by providing comfort or offering reappraisals (e.g., Holodynski et al., 2013; Silkenbeumer et al., 2024; Zinsser et al., 2021). Similarly, attachment theory suggests that secure attachment and corresponding parenting practices and styles, such as sensitive and responsive parenting behavior, and prompt and adequate responses to children's needs, are crucial to develop the ability to openly monitor and acknowledge one's emotions and to adaptively regulate them (Cooke et al., 2019; De Wolff and van IJzendoorn, 1997; Girme et al., 2021; Koehn and Kerns, 2018; Morris et al., 2007).

In the domain of self-regulated learning, Pino-Pasternak and Whitebread (2010) summarized several parenting dimensions and practices conducive to children's acquisition of self-regulated learning. They identify the provision of challenge (encouraging children to face difficulties), autonomy (providing their children choice and showing appreciation for autonomous decision), and contingency (parental responsiveness to children's needs and emotions) as three core dimensions. At these dimensions and their intersections, six parenting practices unfold: Metacognitive talk (as a means to provide and scaffold challenge) means to involve a child in conversations about challenges and to collaboratively work on (strategic) solutions to problems. At the intersection between autonomy and challenge, encouraging active participation means not only to involve children into challenges, but also to give the child autonomy to deal with it. To support autonomy, supporting an understanding of control refers to helping children understand that their own regulatory activities led to success. At the intersection between autonomy and contingency, adult-child shifts in responsibility mean that parental provision of autonomy adapted to the child's current situation and ability as scaffolding aids the acquisition of self-regulation to neither under- nor overchallenge children. Emotional responsiveness as a means of showing contingency broadly means adapted and fine-tuned responses to children's metacognitive activities, emotions, and motivation in dealing with tasks. Last, at the intersection between contingency and challenge, contingent instructional scaffolds refer to adopting support depending on a child's successes and failures. These suggestions are in some ways similar to emotion coaching and co-regulation as they all refer to means of scaffolding, supporting metacognitive activities about inner states, and contingent and appreciative reactions to children's needs.

Comparable processes may be observed regarding the acquisition of motivation regulation. Similar to responsive parenting, emotion coaching and co-regulation, caregivers may attend and respond to children's motivational difficulties, help them understand motivational difficulties, their causes, and underlying needs through verbalizing and discussing them, and offer ways to deal with them (Morris et al., 2007). Over time, children will increasingly regulate their motivation independently (Holodynski, 2006; Silkenbeumer et al., 2024). The extent to which these parenting mechanisms and strategies can be generalized to parental (and potentially, teacher) support of motivation regulation has rarely been tested and requires future research.

### 4.3 Practical implications for supporting motivation regulation development

Since to date, there is little research on how children acquire motivation regulation skills, it is premature to make specific recommendations on how caregivers and teachers can support its development and which aspects of the development of self-regulation they should prioritize. Yet, some tentative suggestions based on theoretical and empirical considerations summarized above may be given. The occurrence of motivational problems (Figure 1, box A) may increase and affect more areas of life as children grow older and environments may not always be sensitive to their motivational needs. Tackling this aspect, adults may support children in developing core motivational constructs, such as identity, interests, values, and goals. This may help children to develop adequate metamotivational knowledge about themselves and tasks they encounter (Figure 1, boxes a1 and a2) and thus select environments matching their needs. This may also prevent unnecessary motivational problems, as well as support them in finding a reason to self-regulate if motivational problems occur, increasing their motivation to self-regulate their motivation (Figure 1, box C). Regarding monitoring and attribution of motivational problems (Figure 1, box B), due to the close connections between motivation and emotion and their regulation (Stockinger et al., 2025), it may be advisable to support children's understanding of their motivational and emotional reactions and potential underlying motivational problems similar to raising emotional awareness and emotion coaching (Silkenbeumer et al., 2024). Also, in accordance to findings regarding the development of emotion regulation, scaffolding children in the problem-solving processes, and coping with these situations may support their acquisition of motivation regulation strategies and knowledge about motivation regulation (Figure 1, boxes D, a3, and b2). To this end, learning from competent role models in real-life or fiction (such as fairy tales) may be helpful as discussed earlier and implemented in emotion regulation trainings (e.g., Seeger and Holodynski, 2022). Thus, adults themselves may be well-advised to acquire and demonstrate adaptive motivation regulation, explicitly demonstrating how they overcome motivational problems at home. Yet, strategies demonstrated should be simple enough in terms of their developmental requirements for children to imitate them, or involve explicit behavioral scaffolding which is reduced over time. At a more distal level, supporting children's cognitive development (for example, of executive functions or language abilities) may also prove beneficial for the more specific motivation regulation skills. However, further research examining the role of these core abilities for the development of motivation regulation is needed. For schools, previous research has shown that self-regulated learning programs are effective at primary school level and that they also impact students' motivation (Dignath et al., 2008). This suggests that other forms of self-regulation training may help students' motivation, but does not reveal to what extent specific support (and which type of support) may be helpful for children to acquire motivation regulation skills. Thus, more longitudinal and intervention research is needed here to explore to what extent and which specific support measures by teachers and caregivers may be helpful to students' development of motivation regulation.

## 5 Factors influencing the development of motivation regulation

In addition to learning mechanisms and environmental influences described above, several developmental psychological prerequisites of the child itself may impact the development of motivation regulation. Among these are cognitive factors including executive functions, sustained attention, theory of mind, and language abilities, as well as contextual factors, such as culture.

### 5.1 Cognitive factors influencing the development of motivation regulation

Executive functions are a group of top-down mental processes necessary to focus attention and direct action in novel situations, including inhibitory control (overriding strong internal urges by controlling actions and thoughts), working memory (the ability to hold information in mind while transforming it mentally), and cognitive flexibility (adjusting to and switching between demands or rules; Diamond, 2013; Figure 1, boxes c1-c2). Executive functions have been linked to the development of a multitude of self-regulatory abilities and wellbeing-related outcomes in later life (Diamond, 2013). For example, childhood executive functions are related to several internalizing and externalizing problems (e.g., Stucke and Doebel, 2024; Yang et al., 2022), several social, health, and behavioral outcomes (e.g., Stucke and Doebel, 2024), and academic achievement (Samuels et al., 2016; Willoughby et al., 2019). Notably, some of this evidence points toward bidirectional relations between outcomes and executive functions and differential results for specific components of executive functions, as well as toward more indirect relations between executive functions and later outcomes via other early skills. It can therefore be assumed that they are also important for the development of motivation regulation in several ways. As already outlined above, sustained attention and working memory capacity are important for holding in mind both the information of one's current goal(s), motivational experience and attributions, current and future goals, and for finding options to regulate them. Cognitive flexibility may be important for switching between different goals, while inhibitory control and sustained attention may help to shield goals from interfering impulses and focus attention on the regulatory process. This implies that specific aspects of executive functions are differentially important for the development of specific components of the motivation regulation process. Yet, executive functions have been found to be relevant for self-regulated learning abilities in preschool children (e.g., Grüneisen et al., 2024).

Sustained attention is defined as the ability to maintain task or goal focus and engagement over extended periods of time, especially during repetitive and monotonous tasks (Unsworth and Robison, 2020), involving several neurophysiological and neural networks (Fortenbaugh et al., 2017). This ability develops during childhood and adolescence with rapid changes around 5 to roughly 10 years, after which only smaller changes are observed (Betts et al., 2006; Guy et al., 2013). Both in children and adolescents, sustained attention is related to school achievement (Gallen et al., 2023; Steinmayr et al., 2010). Because of its critical role for the maintenance of task engagement, deficits in sustained attention may be associated with more frequent or more severe motivational problems, especially effort and opportunity costs, because tasks are experienced as more effortful and take longer. As sustained attention helps to discriminate relevant from irrelevant task stimuli, it may also play a role in motivational monitoring abilities to turn attention toward stimuli relevant for regulation only when really necessary. Vice versa, sustained attention may also benefit from motivation regulation: If children remind themselves of the personal significance of a monotonous task like repeating vocabulary (e.g., reminding themselves how useful knowing the words is for the test) or increase their situational interest in it (e.g., repeating the words in funny voices), they may also find it easier to sustain attention on the task.

Closely intertwined with the development of other cognitive abilities, theory of mind, as the ability to understand and infer mental states of oneself and others, may represent an important precondition for the development of motivation regulation (Carlson and Moses, 2001; Moses and Carlson, 2004; Wellman, 2014; Figure 1, box c4). Several steps in the development of theory of mind may contribute to the development of motivation regulation: An understanding of intentional agency occurs by the end of the first year of life and may build the basis for forming and understanding goals (Wellman, 2011). This understanding develops over the next 3 years as children increasingly use latent constructs, such as desires or beliefs, and form theories about the mental life of themselves and others. With this progression, children's vocabulary becomes more inclusive of words indicating such latent mental states (Wellman, 2011). Additionally, between the second and fourth year of life, children begin to understand diverse desires and, somewhat later, beliefs (e.g., recognizing that two people do not necessarily have the same desires and intentions for the same things across situations or may differ in their perception of the same thing; Wellman and Liu, 2004), which may be a precondition for understanding and forming representations about one's own diversity of desires in the sense of metamotivational knowledge. These shifts in understanding how beliefs and thoughts are related to desires and actions occur around the same time as several improvements in children's general self-regulatory ability and executive functions (Kochanska et al., 2001; Wellman, 2014). This interplay of general cognitive skills (e.g., inhibition, working memory capacity, and mental shifting) and a growing representation of one's own beliefs (as distinct from others) may be important for the development of motivation regulation and “a theory of motivation”, as understanding mental representations about one's motivation and their causes is the foundation for both motivational monitoring and subsequent control.

The development of motivation regulation may also rely on children's abilities to understand and produce language (Cole et al., 2009; Holodynski et al., 2013; Ogren et al., 2024; Figure 1, box c4). Language helps children acquire knowledge about internal states, such as emotions and motivation, e.g., by talking about them with caregivers. It supports mental representations about inner states by labeling them, making room to distance oneself from the experience, and making them accessible to memory and mental transformations (e.g., Harris et al., 2005). Language is also involved in self-regulation, as many strategies to regulate oneself rely on self-talk and inner dialogues (e.g., efficacy self-talk, reminding oneself of one's goals, or reprioritizing; Klinkhammer et al., 2022).

### 5.2 The role of culture in the development of motivation regulation

To date, most studies on motivation regulation have been conducted in the United States and Western Europe, but several studies have been conducted in non-Western countries (Trautner et al., 2025). Yet, as there is also variation within countries, for example, based on ethnicity, explicit investigations of the role of culture in the development of motivation regulation are still missing. Culture refers to a multi-layered concept including real-world objects and subjective aspects, such as norms, values, beliefs, traditions and roles with an impact on individuals' and groups' behaviors (King et al., 2018). Culture is visible not just at a personal level as mental representations and personal behaviors, but also at group level through shared practices, beliefs, and collective actions, and through institutions and the norms and practices they embody (King et al., 2018). Since culture influences motivation in various ways, for example, through constructions of the self in relation to society or valuing of goals through norms, it is likely that some aspects of (the development of) motivation regulation are also influenced by culture, while others may be shared (McInerney et al., 2004; Usher, 2018). For example, the motivational theory of life-span development suggests that control striving as a motivational principle is universal across cultures, as is the functional primacy of primary over secondary control (Heckhausen and Schulz, 1995). Miele et al. (2024) summarize findings demonstrating that while emphasis on specific goals differs somewhat between cultures, peoples' judgments of task-motivation fit appear to be universal. Also, socializers' beliefs, socialization goals, and thus behaviors regarding motivational support toward children may be formed by cultural values and requirements regarding the development of children in general (e.g., Friedlmeier et al., 2011; Keller et al., 2006). This implies that future research on the development of motivation regulation should take cultural influences into account at different levels: For example, at the individual level, culture-dependent subjective constructions of personal relevance and goals may impact which strategies are learned earlier, later, or at all. At the group level, family and classroom practices highlighting goals, providing or avoiding occurrences of motivational challenges, and teaching and parenting practices may be subject to culturally shared norms and values. Finally, at the institutional level, the impact of institutional rules and structures on the acquisition of motivation regulation should be considered.

### 5.3 Developmental sequence and timing of motivation regulation

To date, there is little research explicitly examining how the different aspects of the motivation regulation process develop, which role both individual and contextual factors play in this development, and how motivation regulation builds on broader (cognitive) abilities and contextual factors. This raises the question which developmental preconditions need to be developed to which degree for children to master specific motivation regulation challenges, and around which age they show these abilities. A uniform developmental timeline for the development of the different aspects of the motivation regulation is not possible to date. Yet, the following examples may inspire future research on building such a framework.

An open question to date is whether the acquisition and implementation of a specific motivation regulation strategy may depend on the occurrence of a matching motivational problem earlier in the process. For example, since children experience pleasure and joy or, respectively, displeasure and boredom in tasks rather early, it is likely that they will form mental representations of this problem early on. However, unless they develop a higher order incentive to increase their task enjoyment (such as higher order goals or perceived environmental pressures), they may not acquire strategies to do so. Still, since this motivational problem occurs rather early compared to motivational problems due to low success expectancies resulting from increasingly realistic ability self-concepts (see above), the development of strategies to regulate task value may occur earlier than strategies dealing with motivational problems due to low success expectancies. The underlying assumption here is, however, that strategies are acquired later, after a specific motivational problem is encountered. This may not always be the case though as strategies can be acquired in response to different (non-motivational) challenges and then generalize to motivational problems before they occur. For example, proximal goal setting may be learned as a cognitive strategy to approach complex tasks by planning (Pino-Pasternak and Whitebread, 2010; Pintrich, 2004), including the positive side effect of enhancing success expectancies. Thus, future research examining this assumption regarding the developmental sequence is needed.

Further, more complex and abstract strategies may develop later than simple strategies or simple forms of complex strategies. For example, prioritizing may be visible earlier when there are only two action alternatives which can be selected by simply showing one action first, whereas children may succeed at prioritizing between more than two alternatives later as they require better working memory capacities, language abilities to indicate priorities, and control of action impulses. Such a pattern is supported by the finding that 6-year-olds already showed a good understanding of behavioral strategies, but less of mental strategies (Cooper and Corpus, 2009). This implies that the occurrence of motivation regulation abilities may depend much on the difficulty of the task at hand, as well as on cognitive, motivational, and affective developments during childhood. Figure 1 attempts to capture some of these effects of developmental preconditions on the different aspects of the motivation regulation process, yet, several relations may be missing and require further investigation.

Given the development of major developmental preconditions, such as general cognitive abilities, as well as the development of motivational aspects itself, it is likely that simple forms of motivation control can be observed in 4-to-6-year-olds already, and that given the further continuous improvements in many of these aspects, these developments and refinements continue even beyond childhood. Thus, the development of motivation regulation should not be thought of as a “can or cannot do” concept in children, but a rather gradual process, which depends much on the facet of the process examined, the difficulty and way specific motivational challenges are assessed, and the developmental state of requirements necessary for mastering specific tasks. To spark future research on how exactly which developmental preconditions and contextual factors are associated with the development of different motivation regulation strategies, Table 2 summarizes several preconditions which may be necessary at least at a very basic level for respective strategies to occur.

## 6 Future research questions and recommendations for the development of motivational self-regulation during childhood

The sparse existing evidence on motivation regulation in pre- and primary school children to date suggests that already preschool children monitor and regulate some aspects of motivation. Drawing on theories and evidence on self-regulation from other domains, several specific research questions and hypotheses for future research can be drawn.

### 6.1 Formation and differentiation of motivational problems during childhood

First, 3- to 4-year-olds, and potentially younger children, have representations of short- and long(er)-term goals, as well as possible disruptions or conflicts between these goals that could prevent them from achieving them. However, there has been less systematic research into how effectively children monitor and attribute their motivational problems, which motivational problems they experience and to which causes they attribute them (e.g., low expectation of success, low personal significance, low interest, low usefulness for their own goals, etc.). Therefore, the research question arises how the formation of mental representations of different types of motivational problems and the ability to attribute them to specific causes develops. It can be expected that with a more differentiated motivational self-system regarding interest, identity and ability self-concepts, and goals, more differentiated motivational problems arise. For example, with increasingly higher-level goals and differentiating interests from childhood into adolescence, children may face increasingly complex motivational problems and needs for regulation, such as personally significant tasks which are nonetheless experienced as boring.

### 6.2 Development of strategies for motivational control

This differentiation of motivational problems may be important because previous research suggests that control develops later than monitoring, potentially as a response to experiencing or observing challenges (Bryce and Whitebread, 2012; Engelschalk et al., 2015; Kuhl and Kraska, 1989). Also, most motivation regulation strategies are not universally applicable for all motivational problems and may thus evolve in correspondence to specific problems only (Table 2). Future research is needed to address these questions: Which strategies do children use to regulate their motivation? Does the knowledge (and effective application) of motivation regulation strategies develop later than monitoring of motivational problems? And do strategies develop depending on the experience and/or observation of different motivational problems? Further, it should be examined whether the trend observed in previous research, from concrete behavioral strategies to more abstract mental strategies, can be replicated and how this can be explained (Cooper and Corpus, 2009; Holodynski, 2006; Skinner and Zimmer-Gembeck, 2007). Similar to other areas of self-regulation, it can be assumed that various aspects of motivation regulation also become more effective and efficient with increasing age (Zimmermann and Pinquart, 2019).

### 6.3 Mechanisms of acquisition of motivation regulation and supporting the process

With regard to the acquisition of motivation regulation strategies, various theoretical approaches emphasize social influences. Direct instruction, observational learning, as well as scaffolding-oriented approaches, such as coaching or co-regulation by caregivers or teachers, are mentioned as mechanisms by which self-regulation abilities are acquired from caregivers, teachers, or peers. Future research should therefore examine through which mechanisms and in which social interactions children acquire motivational monitoring and regulation, and how specific they need to be for motivation regulation. As discussed above, many aspects of self-regulation may develop in parallel, such as regulation of emotion, self-regulated learning, and motivation. From this, practical recommendations for caregivers and teachers regarding how to best support their children's development in general and specific to motivation regulation can be drawn in the future, including suggestions which aspects they should prioritize.

### 6.4 Individual and contextual factors influencing the development of motivation regulation

Various individual competences can be theoretically and empirically assumed to impact (the development of) motivation regulation. These pertain to, for example, cognitive prerequisites. Additionally, individual factors such as gender, may have an impact on motivation regulation (Cooper and Corpus, 2009; Gehle et al., 2023; Morris et al., 2007). Future research is needed regarding not only which individual factors influence motivation regulation, but also how these factors influence the development of motivation regulation, both as necessary prerequisites and as fundamental aspects of regulation itself. It can be expected that several (neuro-)cognitive prerequisites (cf. Figure 1, boxes C) may start to develop first and enable later developments of more specific motivation regulation monitoring and control capabilities. Additionally, they may not only directly impact the developmental timing of acquisition, but also moderate how effectively motivation regulation is conducted in later years (e.g., Schwinger et al., 2009). The same may be true for environmental characteristics: Without adequate environments (e.g., providing both motivational challenges and support to overcome them), motivation regulation strategies may not be acquired (or acquired much more slowly). Finally, while some regulatory mechanisms can be assumed as culturally universal (Heckhausen et al., 2010; Heckhausen and Schulz, 1995; Miele et al., 2024), other aspects may differ by cultural influences (e.g., which strategies are legitimate to use to regulate motivation). Future research should therefore delve more deeply into culturally informed theorizing and empirical study designs to investigate environmental influences.

### 6.5 Operationalizations and study designs to assess motivation regulation in children

To investigate these aspects of the development of motivation regulation, most notably, adequate study designs and assessment tools are required to capture the many aspects of motivation regulation, i.e., monitoring and attribution of specific motivational problems, knowledge and successful application of motivation regulation strategies, and forms of co-regulation and increasing self-regulation, as well as developmental trends across time. Research on motivation regulation to date has relied heavily on self-report measures (Trautner et al., 2025), which is adequate when necessary abilities for abstraction and self-reflection on own behavior and thinking and language skills are sufficient (Dörrenbächer-Ulrich et al., 2021; Wolters and Won, 2018). Studies examining motivation regulation during childhood to date have used self-report questionnaires in upper primary school children in ways minimizing such requirements. For example, Ebbes et al. (2024) used self-assessment questionnaire items in an “online” way by connecting them to reflections of a specific task the children worked on, facilitating inferences on one's own behavior. Additionally, diverse other operationalizations depending on the respective aspect of the process investigated have been used. For younger children, however, questionnaires may rely too heavily on general verbal understanding and fluency, reading abilities, and other cognitive demands. This may produce overly positive estimations of their own behaviors (e.g., Gehle et al., 2023).

To investigate beliefs about motivation and knowledge about motivation regulation in children, several studies have used story-based vignettes and interviews (e.g., Cooper and Corpus, 2009; Grüneisen et al., 2024; Gurland and Glowacky, 2011; Jacob et al., 2019; Järvelä et al., 2012). Vignette-based interviews are helpful to assess children's beliefs regarding how motivation works, and which strategies they think may be appropriate. While these forms bypass limited reflection and abstraction capabilities, and can aid in limited (expressive) language abilities, they have three major drawbacks. They do not provide insight regarding whether motivation regulation is stable across situations or whether responses are instead, problem-specific solutions children come up with in the situation. Additionally, they still rely on receptive and expressive language skills to make inferences, and they do usually do not provide information on what the children themselves would to. However, this method may be useful in future research if vignettes with diverse motivational problems are used and do not only rely on written or spoken text, but more strongly on, for example, puppet plays, reducing the amount of language involved further. While puppet play may be engaging and motivation for children in experimental settings, future operationalizations of motivation regulation involving puppets need to ensure children adequately represent and interpret the puppets and their play, that the procedure is culturally sensitive, and that there is sufficient external validity and generalizability to real-world situations (Paulus and Caporaso, 2024).

To assess actual strategy use, observational tools have been used (e.g., Whitebread et al., 2009). In such observational studies, children usually worked on a task, while their behaviors and utterances are coded according to a coding scheme. Such operationalization are advantageous if children have little language abilities and are not yet able to adequately reflect on their behaviors. Since observations are made by others (researchers, parents, teachers), they are less prone to biases if coded in a standardized way. Their disadvantage is that motivation regulation is mainly an internal process and not all aspects of it can be observed in behavior. Thus, many forms of regulation may be missed if not combined with explicit think-aloud instructions.

Overall, while there is certainly not “the” one method to assess motivation regulation during childhood, six major recommendations can be made: For one, operationalizations should, first and foremost, match the aspect of the motivation regulation process examined, for example, beliefs and knowledge about motivation, motivational monitoring, or strategy application. Second, they should not confound motivation regulation (e.g., proximal goal setting, performance self-talk) and motivated behavior (such as task enjoyment, effort expenditure, or time on task) as the result of effective motivation regulation. However, these behavioral effort-related outcomes may be valid operationalizations of effort as one outcome of motivation regulation in younger children compared to self-report of intended effort. Third, the younger the children examined are, the more researchers should take limited self-reflection and language capabilities into account by, for example, reducing the amount of language or the level of reflection of one's behavior. Fourth, combining several methods into mixed methods assessments, for example, combining observational with think-aloud techniques, may yield more valid (yet not always consistent) results. Combining self-report and observation may also help interpret behaviors, which are more visible but not necessarily interpretable in terms of whether they serve motivation regulation or some other goal. For example, proximal goal setting may serve motivation regulation, but may also be used when motivation is high during activity planning. Fifth, the difficulty of specific operationalizations, especially behavioral tasks, should be varied systematically across age groups. Task difficulty is related to developmental preconditions for solving a task and may thus produce different results in different age groups. If tasks are made easier, specific behaviors may be observed in even earlier years, but are trivial to older children. For example, utilizing the strategy “prioritizing” may be easier under task conditions in which there are only two instead of four action options, or if these action options are not equally attractive as opposed to all being equally appealing. Also, even if specific behaviors start to occur around a mean age, there is usually large interindividual variability within age groups. Thus, providing tasks with variable difficulty in different age groups may also allow for better comparisons (for an example of this logic, see Bryce and Whitebread, 2012). Sixth, varying task difficulty is especially important in longitudinal studies for comparing children's performance at tasks across time. To this end, longitudinal studies are needed beyond cohort comparison studies (e.g., Cooper and Corpus, 2009), to track actual developmental changes.

### 6.6 Development of motivation regulation beyond childhood

Since the focus of the current review was on development of motivational self-regulation in childhood, developments in adolescence and across the further life span have not been discussed. Many developmental progresses and trends discussed here, however, will likely continue from childhood to adolescence. Additionally, motivational development itself continues into adolescence and adulthood, with inter- and intraindividual changes in many motivational constructs being observed (e.g., Cole et al., 2001; Gaspard et al., 2020; Jacobs et al., 2002; Orth et al., 2021; Wigfield et al., 2015). Thus, regulation processes may also adapt to the challenges which adolescents experience. Studies to date show that adolescents report a similar repertoire of motivation regulation strategies compared to adults (e.g., Fong et al., 2024; Park, 2022; Villar et al., 2024). In their meta-analysis, Fong et al. (2024) found that associations between some (but not all) motivation regulation strategies and achievement were stronger in primary and secondary school students compared to postsecondary students, which was interpreted as resulting from different environmental characteristics learners face. This demonstrates how not only individual developmental characteristics, but also environments encountered shape motivation regulation. Additionally, motivation regulation may become more important in adolescence and beyond due to increasing intensities of motivational problems and autonomy from parental and teacher support in solving them. Future research should therefore examine such developmental trends beyond childhood in longitudinal studies not only considering age, but individual and environmental characteristics and how they contribute to long-term psychological functioning beyond childhood.

## 7 Limitations of the presented framework

The presented framework has several weaknesses and blind spots. First, there is no assumption regarding the shape of developmental trends, whether it is (non)linear, and whether there is a “mature” level of motivational self-regulation (Adolph et al., 2008; Miller, 2022). This is linked to the question of whether and how motivation regulation develops and changes beyond childhood and adolescence, as well as the question which developments can be classified as adaptive and functional at an individual level and across individuals (Heckhausen and Schulz, 1995; Miele et al., 2024). Second, several proposed components of the model may change simultaneously and dynamically spark changes in other components (Thelen and Smith, 2007). Given such bi-directional co-developments, precisely predicting the timing of specific developments requires longitudinal studies, which not only focus on age-related changes, but also on their dependence on individual and contextual factors as well as interindividual variabilities in developmental trajectories. Third, several important factors influencing the development of motivational self-regulation may be missing. This pertains especially to biological, neurophysiological, and psychological factors more closely tied to general cognitive development. A more differentiated view of societal and cultural factors should therefore be developed further, for example, regarding the question which emphasis culture places on children autonomously setting goals and working toward them in a self-regulated manner (Skinner et al., 2022a,b). Future amendments and empirical tests of the framework should therefore consider such aspects and draw careful conclusions with regard to generalizability of findings across cultures.

## 8 Conclusion

The current review provides an integrative framework and suggestions on how to examine the development of motivation regulation during childhood. It can be concluded that motivation regulation is a process consisting of several aspects from the occurrence of diverse motivational problems to their (successful) regulation. The interplay of the development of these components should be examined in future research. Additionally, the influence of interindividual differences in personal factors, such as cognitive abilities, should be considered to explain the emergence of specific motivation regulation skills. Simultaneously, contextual influences, such as cultural similarities and differences or parental behaviors and beliefs, on the development of motivation regulation should be considered. This also includes the question by which mechanisms children acquire skills specific to motivation regulation. To examine these questions, future studies should focus on mixed methods operationalizations including behavioral observations to validly assess motivation regulation, systematically vary the difficulty of tasks, and compare these tasks in longitudinal studies. Such longitudinal studies are especially needed to examine the impact of early motivation regulation on outcomes later in life.

## Data Availability

The original contributions presented in the study are included in the article/supplementary material, further inquiries can be directed to the corresponding author.
